# Identification of *EPCAM* mutation: clinical use of microarray

**DOI:** 10.1002/ccr3.914

**Published:** 2017-05-10

**Authors:** Queenie K.‐G. Tan, Diana M. Cardona, Catherine W. Rehder, Marie T. McDonald

**Affiliations:** ^1^ Division of Medical GeneticsDepartment of PediatricsDuke University Health SystemDurhamNorth Carolina; ^2^ Department of PathologyDuke University Health SystemDurhamNorth Carolina

**Keywords:** Congenital tufting enteropathy, consanguinity, *EPCAM* mutations, failure to thrive

## Abstract

We report a case of an infant with congenital tufting enteropathy (CTE) who presented with severe failure to thrive despite multiple interventions. This study illustrates that CTE may be missed by endoscopy, and the use of chromosomal microarray and immunohistological analysis may be integral to diagnosis.

## Introduction

Congenital tufting enteropathy (CTE), also known as intestinal epithelial dysplasia (IED), presents in infancy with severe diarrhea and failure to thrive 1. It is a rare disorder with an estimated prevalence of 1 in 50,000 to 1 in 100,000 live births in western Europe 1, but appears to be higher in areas with consanguineous families of Arabic origin 2. Infants develop a watery diarrhea after birth that persists despite bowel rest, requiring parenteral nutrition for growth. The clinical course of patients with CTE can be of varying severity; there have been cases of patients with CTE who have been completely weaned off total parenteral nutrition (TPN) 3 with improvement in their histological lesions. Intestinal transplantation is indicated before the onset of severe complications 4. There are two broad clinical subtypes of CTE: isolated CTE versus CTE associated with syndromic features such as craniofacial dysmorphisms, gastrointestinal malformations (choanal atresia, anal imperforation), hematologic anomalies 5, 6, 7, punctuated keratitis or conjunctivitis 8, arthritis 9, 10, and skeletal dysplasia 7. Histologically, CTE is characterized by villus atrophy, crypt hyperplasia, and focal epithelial tufts typically found in the duodenum and jejunum 11. Histologic studies have suggested that defective epithelial cell interaction has an important pathologic role in CTE 11.

Mutations in the epithelial cellular adhesion molecule (*EPCAM)* gene were identified as causative for CTE 11 using a region of homozygosity found in two affected patients in a consanguineous family, and sequencing candidate genes in the region. Subsequent reports have confirmed the association of mutations in *EPCAM* with CTE 2, 9, 10, 12, 13. *Epcam* knockout mice have also been shown to develop CTE 14. More recently, mutations in *SPINT2*, a serine protease inhibitor thought be involved in epithelial regeneration, have also been associated with CTE 12, 15, 16. It appears that isolated CTE is associated with *EPCAM* mutations, whereas patients with *SPINT2* mutations have the syndromic form of CTE 16.

We report a case here of a patient with *EPCAM*‐associated CTE, who carries two variants at or close to the 5′ end of the gene, which may produce essentially no protein product. These two variants are 5′ to most of the variants reported in literature, excluding large deletions.

## Case Report

The patient was first brought to medical attention at age 2 weeks for weight loss and vomiting. He was born full term with appropriate birthweight of 3430 g. Pregnancy and delivery were reportedly uncomplicated, and he was discharged home with his parents on the second day of life. He was initially breastfed but had recurrent emesis and continued weight loss, despite formula change and treatment for gastroesophageal reflux. While hospitalized multiple times for failure to thrive, he was noted to have nonbloody and nonbilious emesis and large, loose stools as well as episodes of hematochezia. Stool studies showed elevated osmolality and osmolar gap (but normal electrolytes tested), slightly low pancreatic elastase, high fecal fat, positive reducing substances and mildly elevated calprotectin, overall consistent with malabsorption. Weight loss persisted despite being on full‐calorie nasogastric feeds with different formulae, including amino acid‐based formula and carbohydrate‐free formula. He was noted to have a nonanion gap metabolic acidosis thought to be secondary to bicarbonate loss and severe dehydration and was started on bicitra. Transaminases were also elevated, suspected due to malnutrition. Imaging for pyloric stenosis and malrotation was negative. Echocardiogram and head ultrasound were normal. Abdominal ultrasound was only notable for nephrocalcinosis and gallbladder sludge. He eventually underwent gastrostomy tube placement and was started on TPN for supplemental nutrition with continued poor weight gain.

On examination, he was noted to have minimal subcutaneous fat, with a prominent forehead, high nasal bridge, and pointed chin, with no striking dysmorphology noted.

Family history was significant for consanguinity in the parents (second cousins), with two prior miscarriages from the union and a healthy older brother. The family history was negative for individuals with similar clinical presentation.

**Figure 1:**
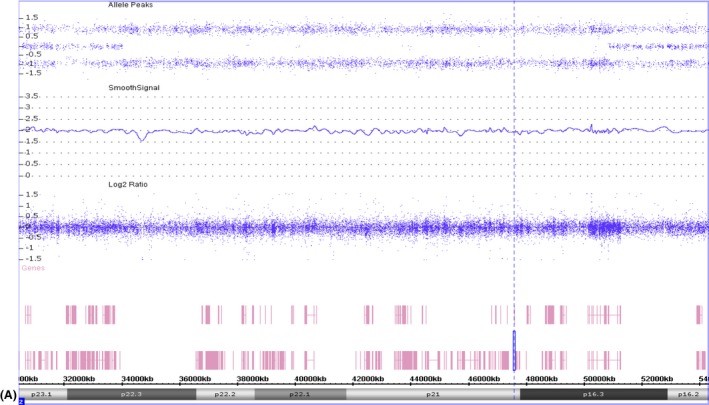
Microarray analysis showed several independent regions of homozygosity (ROH) encompassing >12% of the genome. a) A 16‐Mb ROH on chromosome 2 (chr2:34,008,052‐50,861,049) demonstrating a copy number state of 2 and no heterozygous SNPs. The dotted vertical line denotes the location of the *EPCAM* gene.

Metabolic workup was only remarkable for moderate elevation of lactate on urine organic acids and mildly elevated alanine on plasma amino acids. Chromosomal microarray (Duke Clinical Cytogenetics Laboratory) showed several independent regions of homozygosity encompassing more than 12% of the genome (Fig. 1). While this percentage is greater than the percentage observed in second cousin matings in an outbred population (~1.5%), it is within the range typically observed in families with multiple loops of consanguinity. Searching for autosomal recessive disorders associated with failure to thrive and diarrhea in the blocks of homozygosity of more than 3 Mb revealed a list of ten genes (*EPCAM, DCLRE1C, NEUROG3, SLC12A1, PMM2, CIITA, SCNN1B, SCNN1G, IL21R, and HSD3B7.)*

**Figure 2:**
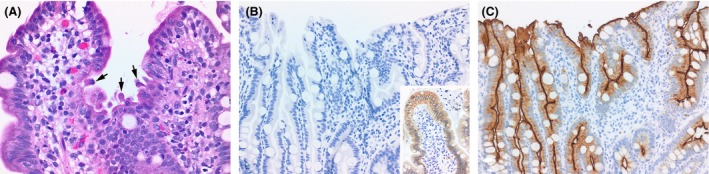
(A) Small intestinal mucosa with small protruding tufts of epithelial cells. No intraepithelial lymphocytosis, significant villous atrophy, or other inflammatory injury is present (H&E, 200x). (B) Immunohistochemistry for EPCAM/MOC31 is negative within the small intestinal mucosa (200x); positive control within the inset. (C) CD10 immunoreactivity is intact, highlighting the normal brush border of the enterocytes (200x).

**Figure 3:**
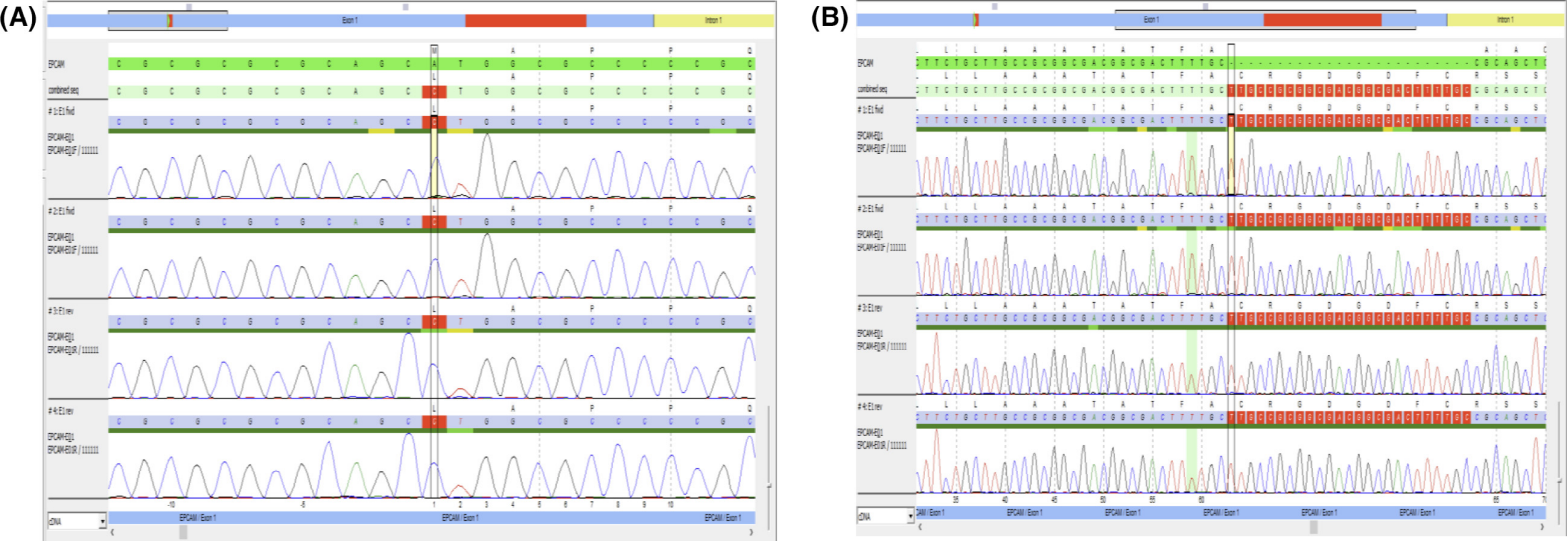
Identification of the c.1A>C variant (A) and the c.38_62 duplication (B) in the *EPCAM* gene.

An esophagogastroduodenoscopy (EGD) was performed prior to review of chromosomal microarray results, with normal gross appearance of the upper gastrointestinal system. Based on microarray data, the possibility of *EPCAM*‐related CTE was raised, and the patient's tissues from endoscopy were re‐evaluated by pathology. Histopathology of duodenal specimens revealed mild reactive epithelial change with increased epithelial apoptosis, mild villous atrophy, and focal areas suspicious for epithelial tufting. Immunohistochemistry for MOC31 (EpCam) was absent in the epithelium (Fig. 2). Sanger sequencing of the *EPCAM* gene (Prevention Genetics) revealed a homozygous, pathogenic sequence variant c.38_62dup (p.Ala22Cysfs*17), which is predicted to result in a frameshift and premature protein termination. He was also found to be apparently homozygous for a sequence variant at the initiation codon c.1A>C (p.Met1?), which is classified as a variant of unknown clinical significance by the laboratory (Fig. 3).

**Figure 4:**
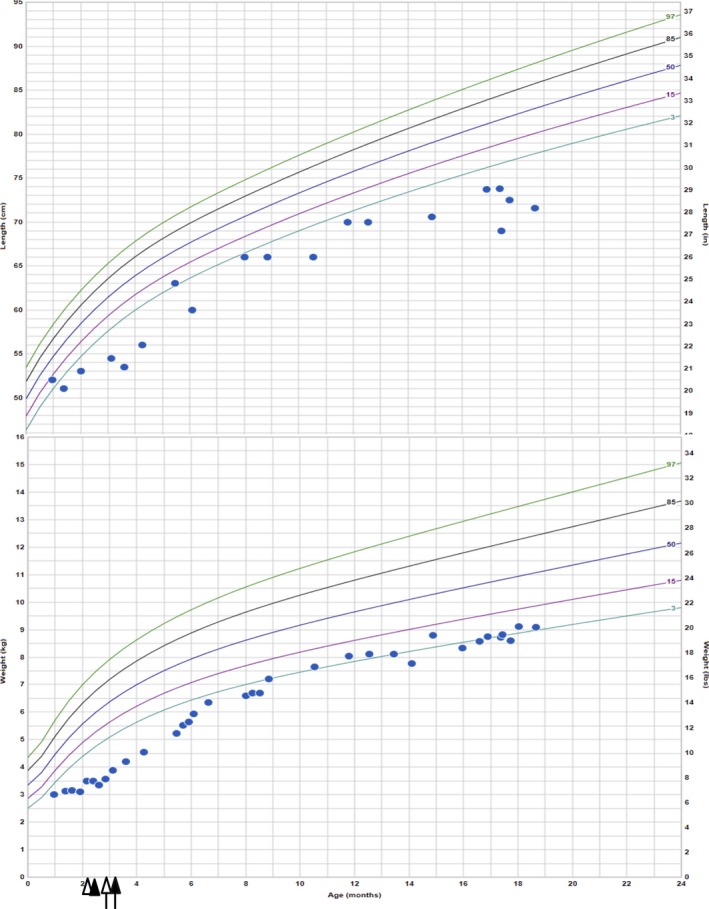
Patient's weight and height plotted on growth chart (World Health Organization). Open arrowhead: EGD performed and chromosomal microarray resulted; closed arrowhead: immunohistological analysis on tissues obtained from endoscopy showed absence of EPCAM staining; open arrow: patient placed on full TPN and enteral feeds stopped; closed arrow: DNA sequencing resulted.

Based on his diagnosis of CTE, all enteral feeds were stopped (except for small amounts of baby food) and he was continued exclusively on TPN for nutrition. He has been gaining weight on TPN (Fig. 4) but has unfortunately had a few line infections. Discussion has been initiated for small bowel transplantation if diarrhea persisted. Nephrocalcinosis has resolved on repeat abdominal ultrasound.

## Methods

### Consent

Informed consent for this case report was obtained in writing from the patient's father.

### Chromosomal microarray

Chromosomal Microarray Analysis (CMA SNP) was performed by the Duke Clinical Cytogenetics Laboratory using the Affymetrix Cytoscan HD array. This array consists of nearly 2.7 million genetic markers incorporating 743,304 single nucleotide polymorphism (SNP) probes as well as 1,953,246 nonpolymorphic copy number variation (CNV) probes with a median spacing of 0.88 kb.

### Histology and Immunohistochemistry

Endoscopically obtained biopsy samples were formalin fixed and paraffin embedded. Five‐micron‐thick sections were stained with hematoxylin and eosin. An additional section underwent immunohistochemical analysis with anti‐EPCAM antibody [ready‐to‐use Dako clone BER‐EP4].

### DNA sequencing

Sanger sequencing of the *EPCAM* gene was performed clinically by Prevention Genetics (Marshfield, WI). Each coding exon of *EPCAM* and about 20 bp of adjacent noncoding sequences were amplified and Sanger sequenced.

## Discussion

We present a case of a patient, born of consanguineous parents, with *EPCAM‐*associated CTE who presented in the neonatal period with emesis, severe diarrhea, and failure to thrive. Mutations in *EPCAM* as the possible cause for his symptoms were considered after a SNP chromosomal microarray identified the *EPCAM* gene within one of the regions of homozygosity. Following correlation with microarray data, histological analysis of duodenal biopsies performed at age 2 months demonstrated focal areas of epithelial tufting. Epithelial tufting has been reported to be difficult to find in the first few months of life 1, 16. While early endoscopy for patients with persistent failure to thrive may be warranted and may reveal other gastrointestinal pathologies, our experience cautions clinicians that patients with congenital diarrhea who have had normal endoscopy results may in fact have CTE, and repeated endoscopies at later ages may be required to observe epithelial tufting. In the absence of obvious pathologic findings, we suggest that immunohistochemical staining for EPCAM should be performed in tissues obtained endoscopically from patients with a strong clinical suspicion for CTE. Molecular confirmation of *EPCAM* pathogenic variants can then be sought if immunohistochemical or pathological findings are suggestive of EPCAM‐related CTE.

We note that calprotectin was mildly elevated in the patient's stool sample, consistent with mild inflammation which could be seen in tufting enteropathy 1, 17, although severe inflammation is typically not associated with CTE.

Our patient's frameshift duplication is located at the end of the signal peptide for EPCAM (amino acid residues 1‐23) and adds 16 novel residues after residue 21 before terminating in a stop codon. This mutation is 5′ to most of the mutations reported in literature and thus essentially produces no protein product. Sequencing also revealed a homozygous variant which is believed to disrupt the *EPCAM* start codon, classified as a variant of unknown significance by the performing laboratory. However, this variant could potentially be pathogenic as well, leading to translation initiation from a downstream ATG which may or may not yield a functional protein. The presence of either of these two variants may explain the severe phenotype of our case. Most reported CTE‐associated *EPCAM* mutations are located in exons 3, 4, or 5, and the protein product is predicted to be missing part of the extracellular domain and/or transmembrane domain 13, and some whole exon/whole gene deletions have been reported 16. Mutations associated with CTE appear to lead to loss of cell surface EPCAM protein 18, 19, which is consistent with immunohistological findings in this case.

Our case here illustrates how we used microarray data to search for genes within the areas of homozygosity possibly associated with the phenotype, then confirmed the diagnosis with histopathological methods and gene sequencing. Chromosomal microarray is frequently used in the genetic evaluation of patients with abnormal growth 20. In patients with consanguineous heritage, SNP microarrays could be used to look for genes associated with autosomal recessive conditions related to the clinical phenotype within regions of homozygosity. For example, in our case, ten candidate genes in the regions of homozygosity were associated with failure to thrive. Clinical assessment of phenotypes associated with pathogenic variants in these genes was crucial in helping us focus on the most likely gene implicated in the patient's disorder, because while many disorders can cause failure to thrive, some of them are associated with other clinical features absent in our patient. We do recommend further testing, including sequencing the implicated genes to look for variants, to confirm the association with the patient's phenotype. This case emphasizes that judicious use of cytogenetics data can help to guide diagnosis, sometimes obviating the need to perform more expensive and time‐consuming genetic tests.

## Authorship

QKGT: helped in drafting the manuscript, analysis, and interpretation of data. DMC: contributed to acquisition, analysis and interpretation of data, and critical revision of manuscript. CWR: helped in acquisition, analysis and interpretation of data, and critical revision of manuscript. MTM: contributed to analysis and interpretation of data, and critical revision of manuscript.

## Conflict of Interest

None declared.

## References

[1] Goulet, O. , J. Salomon , F. Ruemmele , N. Patey‐Mariaud de Serres , and N. Brousee . 2007 Intestinal epithelial dysplasia (tufting enteropathy). Orphanet J. Rare Dis. 2:20. 17448233 10.1186/1750-1172-2-20 PMC1878471

[2] Salomon, J. , Y. Espinosa‐Parrilla , O. Goulet , W. Al‐Qabandi , P. Guique , D. Canioni , et al. 2011 A founder effect at the *EPCAM* locus in congenital tufting enteropathy in the Arabic gulf. Eur. J. Med. Genet. 54:319–322. 21315192 10.1016/j.ejmg.2011.01.009

[3] Lemale, J. , A. Coulomb , B. Dubern , S. Boudjemaa , S. Viola , P. Josset , et al. 2011 Intractable diarrhea with tufting enteropathy: a favorable outcome is possible. J. Pediatr. Gastroenterol. Nutr. 52:734–739. 21478758 10.1097/MPG.0b013e31820731db

[4] Paramesh, A. S. , T. Fishbein , A. Tschernia , N. Leleiko , M. S. Magid , G. E. Gondolesi , et al. 2003 Isolated small bowel transplantation for tufting enteropathy. J. Pediatr. Gastroenterol. Nutr. 36:138–140. 12500010 10.1097/00005176-200301000-00026

[5] Bird, L. M. , M. Sivagnanam , S. Taylor , and R. O. Newbury . 2007 A new syndrome of tufting enteropathy and choanal atresia, with ophthalmologic, hematologic and hair abnormalities. Clin. Dysmorphol. 16:211–221. 17786112 10.1097/MCD.0b013e328274264b

[6] Abely, M. , G. F. Hankard , J. P. Hugot , J. P. Cezard , M. Peuchmaur , and J. Navarro . 1998 Intractable infant diarrhea with epithelial dysplasia associated with polymalformation. J. Pediatr. Gastroenterol. Nutr. 27:348–352. 9740211 10.1097/00005176-199809000-00016

[7] El‐Matary, W. , A. M. Dalzell , G. Kokai , and J. E. Davidson . 2007 Tufting enteropathy and skeletal dysplasia: is there a link? Eur. J. Pediatr. 166:265–268. 16900309 10.1007/s00431-006-0231-z

[8] Roche, O. , M. Putterman , J. Salomon , F. Lacaille , N. Brousse , O. Goulet , et al. 2010 Superficial punctate keratitis and conjunctival erosions associated with congenital tufting enteropathy. Am. J. Ophthalmol. 150:116–121. 20447614 10.1016/j.ajo.2010.01.034

[9] Al‐Mayouf, S. M. , N. Alswaied , F. S. Alkuraya , A. AlMehaidib , and M. Faqih . 2009 Tufting enteropathy and chronic arthritis: a newly recognized association with a novel *EpCAM* gene mutation. J. Pediatr. Gastroenterol. Nutr. 49:642–644. 19820410 10.1097/MPG.0b013e3181acaeae

[10] Ko, J. S. , J. K. Seo , J. O. Shim , S. H. Hwang , H. S. Park , and G. H. Kang . 2010 Tufting enteropathy with *EpCAM* mutations in two siblings. Gut. Liv. 4:407–410. 10.5009/gnl.2010.4.3.407 PMC2956358 20981223

[11] Sivagnanam, M. , J. L. Mueller , H. Lee , Z. Chen , S. F. Nelson , D. Turner , et al. 2008 Identification of EpCAM as the gene for congenital tufting enteropathy. Gastroenterology 135:429–437. 18572020 10.1053/j.gastro.2008.05.036 PMC2574708

[12] Sivagnanam, M. , T. Schaible , R. Szigeti , R. H. Byrd , M. J. Finegold , S. Ranganathan , et al. 2010 Further evidence for *EpCAM* as the gene for congenital tufting enteropathy. Am. J. Med. Genet. Part A 152A:222–224. 20034091 10.1002/ajmg.a.33186 PMC6691968

[13] Thoeni, C. , A. Amir , C. Guo , S. Zhang , Y. Avitzur , Y. M. Heng , et al. 2014 A novel nonsense mutation in the *EpCAM* gene in a patient with congenital tufting enteropathy. J. Pediatr. Gastroenterol. Nutr. 58:18–21. 24048167 10.1097/MPG.0000000000000106

[14] Guerra, E. , R. Lattanzio , R. La Sorda , F. Dini , G. M. Tiboni , M. Piantelli , et al. 2012 *mTrop1/Epcam* knockout mice develop congenital tufting enteropathy through dysregulation of intestinal E‐cadherin/β‐catenin. PLoS One 7:e49302. 23209569 10.1371/journal.pone.0049302 PMC3509129

[15] Slae, M. A. , M. Saginur , R. Persad , J. Yap , A. Lacson , J. Salomon , et al. 2013 Syndromic congenital diarrhea because of the *SPINT2* mutation showing enterocyte tufting and unique electron microscopy findings. Clin. Dysmorphol. 22:118–120. 23689399 10.1097/MCD.0b013e328361d42f

[16] Salomon, J. , O. Goulet , D. Canioni , N. Brousse , J. Lemale , P. Tounian , et al. 2014 Genetic characterization of congenital tufting enteropathy: epcam associated phenotype and involvement of *SPINT2* in the syndromic form. Hum. Genet. 133:299–310. 24142340 10.1007/s00439-013-1380-6

[17] Gerada, J. , J. DeGaetano , N. J. Sebire , S. Hill , M. Vassallo , and T. M. Attard . 2013 Mucosal inflammation as a component of tufting enteropathy. Immunogastroenterology 2:62–67.

[18] Schnell, U. , J. Kuipers , J. L. Mueller , A. Veenstra‐Algra , M. Sivagnanam , and B. N. G. Giepmans . 2013 Absence of cell‐surface EpCAM in congenital tufting enteropathy. Hum. Mol. Genet. 22:2566–2571. 23462293 10.1093/hmg/ddt105 PMC3674798

[19] Ranganathan, S. , L. A. Schmitt , and R. Sindhi . 2014 Tufting enteropathy revisited: the utility of MOC31 (EpCAM) immunohistochemistry in diagnosis. Am. J. Surg. Pathol. 38:265–272. 24418860 10.1097/PAS.0000000000000106

[20] Henderson, L. B. , C. D. Applegate , E. Wohler , M. B. Sheridan , J. Hoover‐Fong , and D. A. S. Batista . 2014 The impact of chromosomal microarray on clinical management: a retrospective analysis. Genet. Med. 16:657–664. 24625444 10.1038/gim.2014.18

